# The role of irreversible pan-HER tyrosine kinase inhibitors in the treatment of HER2-Positive metastatic breast cancer

**DOI:** 10.3389/fphar.2023.1142087

**Published:** 2023-03-02

**Authors:** Zihong Wu, Jiamei Wang, Fengming You, Xueke Li, Chong Xiao

**Affiliations:** Hospital of Chengdu University of Traditional Chinese Medicine, Chengdu, China

**Keywords:** HER2-positive metastatic breast cancer, irreversible pan-HER tyrosine kinase inhibitors, failure of multiple lines of treatment, clinical trials, salvage therapy

## Abstract

Human epidermal growth factor receptor 2 (HER2)-positive metastatic breast cancer (MBC) is the leading cause of cancer death in women. For patients with HER2-positive MBC, after the failure of multiple lines of treatment, there is no optimal line of therapy. A series of clinical trials confirmed that treatment with irreversible pan-HER tyrosine kinase inhibitors (TKIs) in combination with chemotherapy significantly improves patients’ survival outcomes. This review focuses on the pathogenesis of HER2-positive breast cancer, current standard treatments, mechanisms of approved irreversible TKIs, and key clinical trials. The available findings suggest that irreversible pan-HER TKIs, such as pyrotinib and neratinib, in combination with chemotherapy, represent a beneficial salvage therapy for patients with HER2-positive MBC with manageable toxicity. However, further studies are needed to assess the efficacy and safety of this combination therapy.

## Graphical Abstract

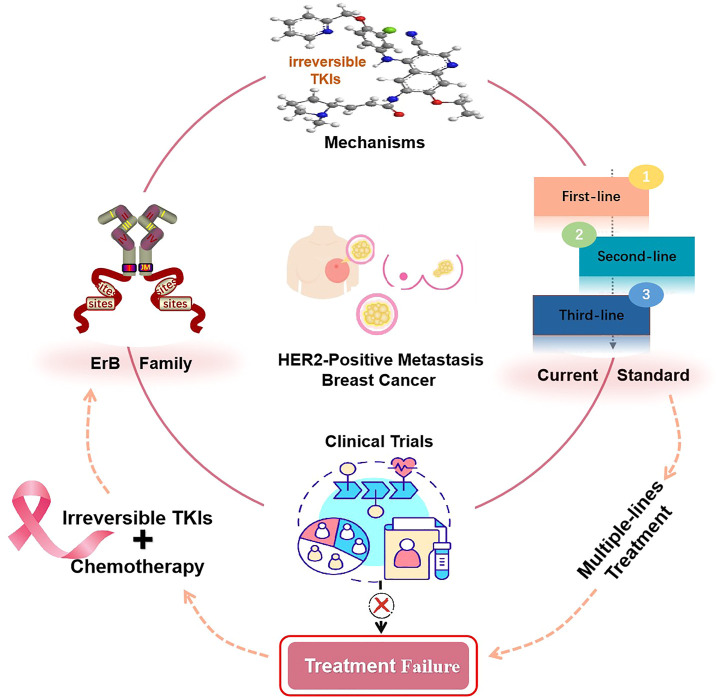

## Introduction

Breast cancer (BC) is the malignancy with the highest incidence, and it represents the leading cause of cancer death in women (Nader-Marta et al., 2022). Approximately 297,790 females in the United States will be diagnosed with breast cancer in 2023, and more than 115 people die each day from breast cancer (Siegel et al., 2023). As women’s social status is increasing, so are the physical and psychological pressures they are under, which is one of the reasons for the rising incidence of breast cancer. Human epidermal growth factor receptor 2 (HER2)-positive breast cancer accounts for about 15%–20% of all breast tumors (Choong et al., 2020). Patients with HER2-positive BC are more likely to experience recurrence and metastasis, especially brain metastasis, leading to poor survival outcomes (Oliveira et al., 2020; Olga Mart ´ınez-S ´aez and Aleix Prat, 2021). HER2-positive metastatic breast cancer (MBC), after failure of multiple lines of treatment, has no effective solution.

Standard anti-HER2 drugs, especially those large in molecular size (pertuzumab, trastuzumab, etc.), show limited diffusion into the brain parenchyma due to the tight junctions of the blood-brain barrier (BBB) and its heterogeneous permeability to small and large molecules (Arvanitis et al., 2020; Bailleux et al., 2021). Numerous clinical trials have confirmed that tyrosine kinase inhibitors (TKIs), which are small molecules, are promising anti-HER2 agents for MBC, having the advantages of oral administration, multi-targeting, and low toxicity (Xuhong et al., 2019). For instance, lapatinib, the first reversible pan-HER TKI, has been approved for HER2-positive MBC, but it is toxic and results in limited improvement outcomes (Oliveira et al., 2020; Schlam and Swain, 2021).

Efforts are underway to develop irreversible pan-HER TKIs, such as pyrotinib, neratinib, and afatinib, to overcome drug resistance partially caused by HER2 receptor mutations. Patients with HER2-positive MBC are usually treated with chemotherapy-based regimens. The PHOEBE trial (Xu et al., 2021) and NALA trial (Saura et al., 2020) showed that treatment with pyrotinib or neratinib combined with capecitabine significantly prolongs progression-free survival (PFS) of patients with HER2-positive MBC compared with the use of lapatinib plus capecitabine, making these some of the current options after failure of multiple lines of anti-HER2 therapy. This review focuses on the pathogenesis of HER2-positive breast cancer, current standard treatments, the mechanisms of approved irreversible TKIs, key clinical trials, and the activity of irreversible TKIs in brain metastases to provide scientific evidence on the regimen of irreversible pan-HER TKIs plus chemotherapy for HER2-positive MBC patients.

## Overview of the market

### HER2-positive breast cancer

**FIGURE 1:**
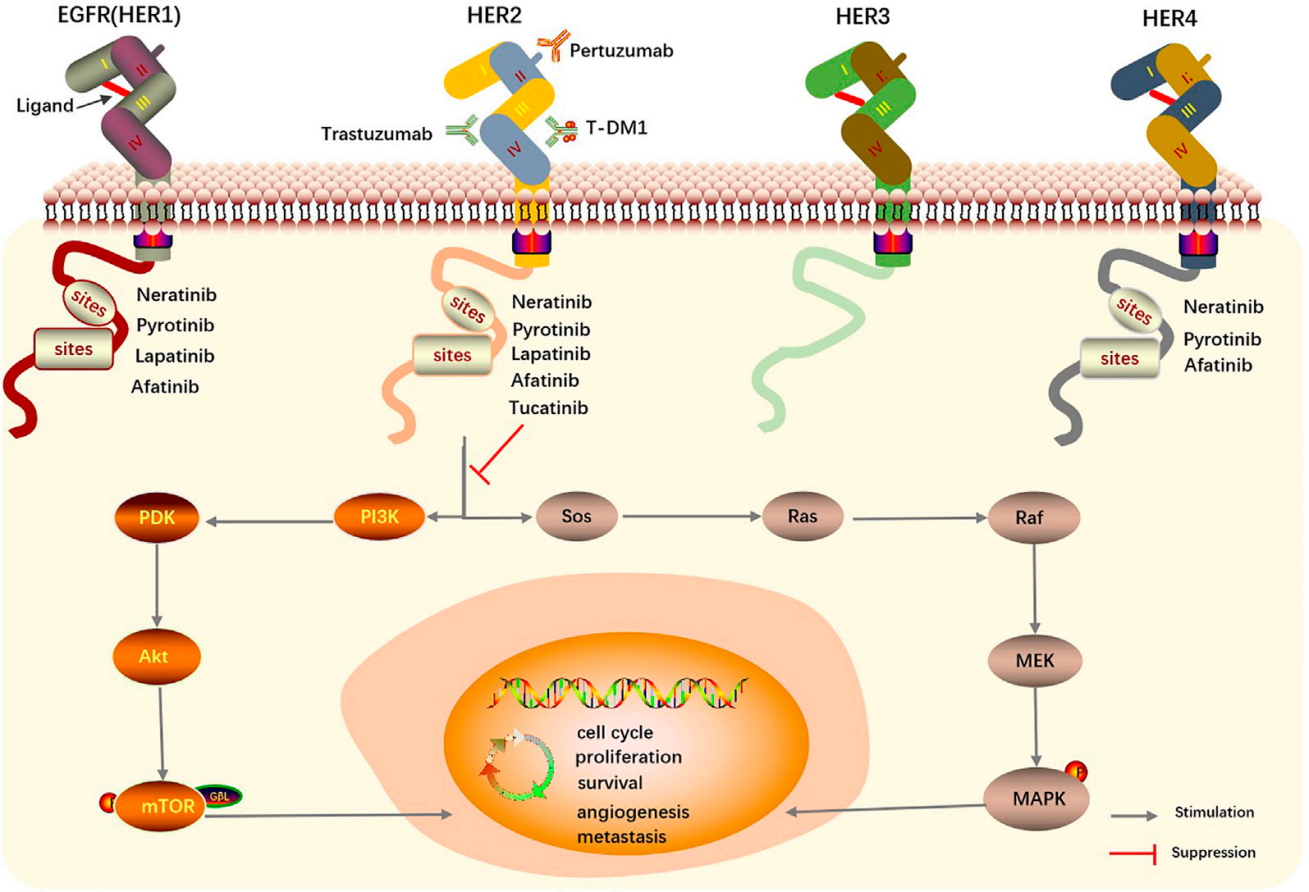
Mechanism of HER2 targeted drugs and EGFR family in breast cancer. Homo- or heterodimers of HER2 lead to the phosphorylation of tyrosine kinase residues in domain IV and regulate various downstream signaling pathways. HER, Human epidermal growth factor receptor. EGFR, epidermal growth factor receptor. T-DM1, trastuzumab emtansine.

Breast cancer overexpressing HER2 has aggressive behavior and is associated with a poor prognosis. HER2(ErbB2) is a transmembrane protein encoded by the oncogene ErbB2 and located on the long arm of chromosome 17 (Spector and Blackwell, 2009; Xuhong et al., 2019). HER2 is part of the epidermal growth factor receptor (EGFR/Erb) family of tyrosine kinase receptors, which consists of four members-EGFR/HER1/ErbB1, HER2/ErbB2, HER3/ErbB3, and HER4/ErbB4-and is closely related to cell proliferation, differentiation, migration, and cancer development (Roskoski, 2019; Sabbah et al., 2020). The extracellular domains of these receptors consist of four components (I-IV). Domains I and III are involved in ligand binding (except ErbB2/HER2), domain II takes part in dimer formation, and domain IV (the carboxyterminal tail) contains several tyrosine phosphorylation sites (Roskoski, 2019). It is generally believed that the HER2 extracellular domain has no known ligand due to its constitutively active conformation and is activated primarily through forming homo- or heterodimers. These dimers lead to phosphorylation of tyrosine kinase residues in domain IV, which regulate various downstream signaling pathways, for example, by activating phosphoinositide 3-kinase (PI3K)/Akt, mitogen-activated protein kinase (MAPK), and Janus kinase (JAK)/signal transducer and activator of transcription (STAT) pathways (Figure 1) (Butti et al., 2018; Xuhong et al., 2019; Schlam and Swain, 2021). Activation of these downstream signal leads to cancer cell proliferation, survival, angiogenesis, cancer stem cell properties, metastasis, and drug resistance (Butti et al., 2018; Wen et al., 2019; Shao et al., 2021).

## Current standard treatments

**FIGURE 2:**
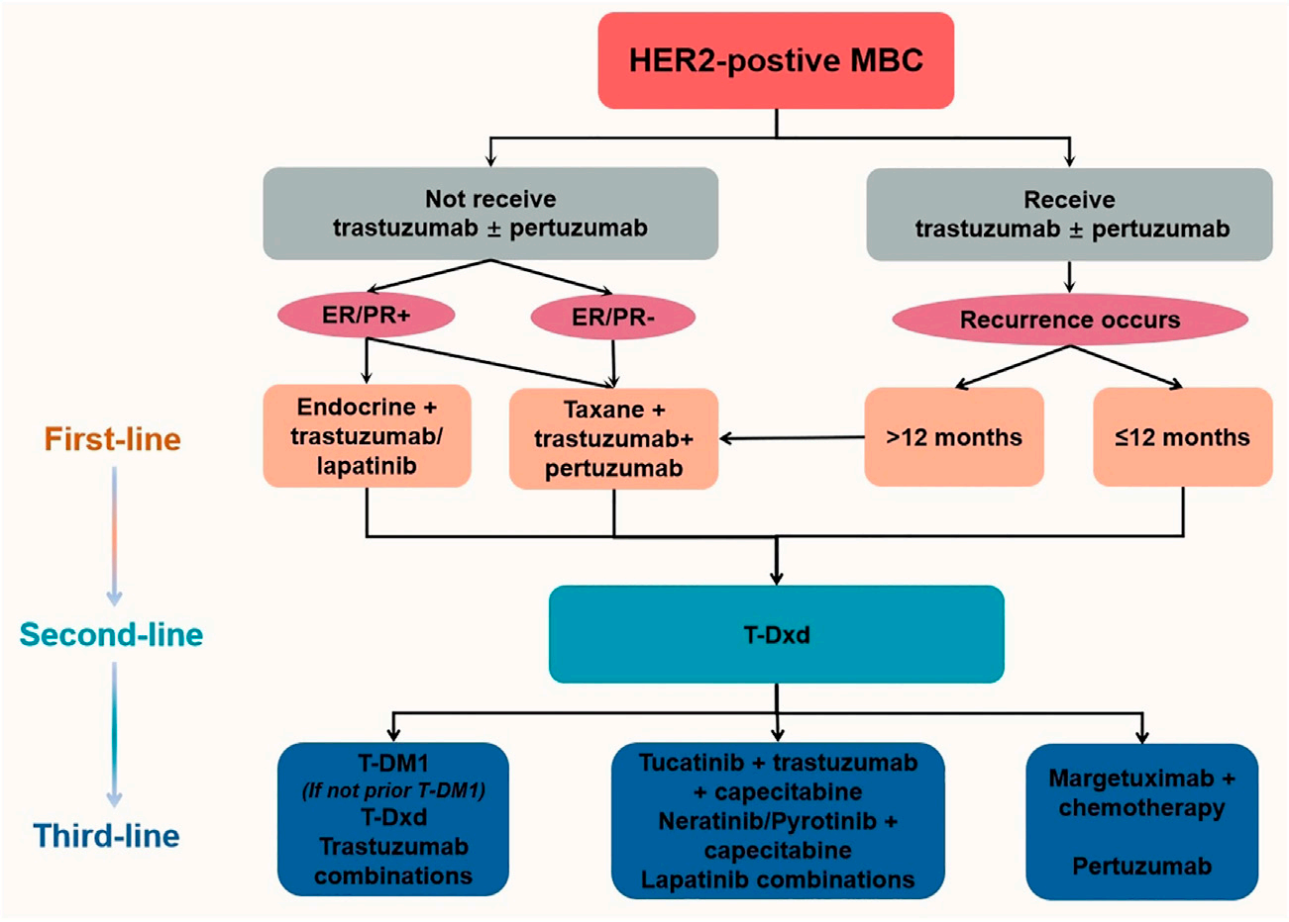
Recommendations for HER2-positive MBC (Giordano et al., 2022). After the failure of multiple lines of treatment, there is insufficient evidence to recommend one regimen over another due to the lack of head-to-head trials. HER2, Human epidermal growth factor receptor 2. T-DM1, trastuzumab emtansine. T-Dxd, trastuzumab deruxtecan.

HER2-positive BC patients are more likely to experience metastasis, but at the same time, they have access to targeted therapies. According to the CLEOPATRA (Swain et al., 2020) and PERUSE trials (Bachelot et al., 2019), the combination of taxane, trastuzumab, and pertuzumab is the current standard first-line treatment for trastuzumab-sensitive, HER2-positive MBC patients. The long-term safety and cardiac safety of docetaxel, trastuzumab, and pertuzumab were demonstrated in the overall population after a median 8-year follow-up period (Swain et al., 2020). After progression on first-line trastuzumab therapy, continued inhibition of the HER2 pathway may provide a survival benefit, and therefore, it is recommended that second-line anti-HER2-targeting agents continue to be used, with trastuzumab deruxtecan (T-Dxd) being the preferred regimen (Giordano et al., 2022). Other options include trastuzumab emtansine (T-DM1), and the combination of TKIs with chemotherapy (Li and Jiang, 2022). Based on a pivotal phase-III trial, the use of lapatinib in combination with capecitabine improves PFS in patients with HER2-positive MBC but has no significant impact on OS compared with using capecitabine alone (Geyer et al., 2006). The results of the influential EMILIA trial (Dieras et al., 2018) confirm the significant PFS and OS benefits with single-agent T-DM1 compared with lapatinib in combination with capecitabine, making this regimen one of the standard international second-line anti-HER2 treatment options. For those with HER2-positive, hormone receptor (HR)-positive recurrent MBC, priority is given to anti-HER2 in combination with chemotherapy or endocrine therapy (Figure 2) (Hua X. et al., 2022).

Although first- and second-line treatment regimens based on phase-III randomized controlled trials (RCTs) are well established, there is still no standard regimen for patients with HER2-positive advanced breast cancer who progress during or after second-line or later HER2-targeted therapy (resistant to trastuzumab/pertuzumab and T-DM1). The latest ASCO guidelines recommend that clinicians should recommend anti-HER2-based therapy as third-line, or further deprioritized, treatment (Giordano et al., 2022). Some reversible pan-HER TKIs, such as lapatinib and tucatinib, will lose their therapeutic effect and encounter resistance when HER2 loses its cytoplasmic antibody binding site for its mutation or truncation (Huang et al., 2020). Overall, after the failure of multiple lines of treatment, there is insufficient evidence to recommend one regimen over another due to the lack of head-to-head trials.

Based on the significant phase-III clinical trials (PHOEBE (Xu et al., 2021) and NALA (Saura et al., 2020)), irreversible pan-HER TKIs, such as pyrotinib and neratinib, in combination with capecitabine, may provide a survival benefit for patients with HER2-positive MBC, making this an option after failure of multiple lines of anti-HER2 therapy. Therefore, this paper explores the implications of using irreversible TKIs in combination with chemotherapy for the treatment of HER2-positive MBC. Next, attention is first focused on the mechanisms of irreversible TKIs currently approved for HER2-positive MBC.

## Mechanisms of approved irreversible TKIS

Three irreversible pan-HER TKIs are currently approved for the treatment of HER2-positive MBC: pyrotinib, neratinib, and afatinib.

### Pyrotinib

Pyrotinib (SHR-1258), an oral irreversible pan-HER TKI, was first conditionally approved in China in 2018 in combination with capecitabine for the treatment of patients with HER2-positive advanced or metastatic BC (Xuhong et al., 2019). It is a small molecule with activity against HER1(IC50: 5.6 nM), HER2(IC50: 8.1 nM), and HER4 (Ma et al., 2017). Studies have suggested that pyrotinib is a potent and selective EGFR/HER2 dual inhibitor that can effectively inhibit the proliferation of HER2+ BC cells *in vivo/in vitro* (Li et al., 2017). By binding with the ATP-binding site of the intracellular kinase region, pyrotinib inhibits the formation and autophosphorylation of the ErbB family homodimers or heterodimers (Xuhong et al., 2019). Another *in vitro* experiment confirmed that pyrotinib significantly inhibits the proliferation, invasion, and migration of breast cancer cells. It induces G1 phase cell cycle arrest and downregulates the expression of p-p65, p-Akt, and FOXC1. That is, pyrotinib primarily exerts its potent anti-tumor effects by blocking the activation of the Ras/MAPK and PI3K/AKT signaling pathways (Wang C. et al., 2021).

### Neratinib

Neratinib (HKI-272) is an oral, irreversible inhibitor of HER1, HER2 and HER4 (Schlam and Swain, 2021). Neratinib is the only TKI currently approved by the US FDA for early-stage HER2+ BC and was approved in 2017 (Deeks, 2017). The SUMMIT trial (Hyman et al., 2018) demonstrates that neratinib exhibits maximum activity in patients with HER2 mutant breast cancer, with missense mutations involving extracellular and kinase domains, as well as kinase domain insertions. Based on NEfERT-T (Awada et al., 2016), NALA (Saura et al., 2020), and TBCRC022 (Freedman et al., 2016) trials, neratinib was approved by the US FDA, in combination with capecitabine, for patients with HER2+ MBC who received at least two lines of anti-HER2 therapy (Schlam and Swain, 2021). By covalently binding to Cys-773/805, a cysteine residue in the ATP-binding pocket of the ErbB family (HER1/2/4), neratinib inhibits the activation of the extracellular signal-regulated kinase (ERK)/MAPK and PI3/Akt pathways and reduces the expression of cyclin D1, while upregulating the level of p27, ultimately leading to cell-cycle arrest and decreased proliferative viability of cancer cells (Booth et al., 2020; Smith et al., 2021). The IC50 values of neratinib required to inhibit the receptor kinase EGFR, HER2 and HER4 activity were 92, 59, and 19 nM, respectively (Kourie et al., 2016). Neratinib can also inactivate PI3K and ERK signaling by degrading MST4 through autophagy (Dent et al., 2020). Neratinib not only effectively blocks the dimers of HER2 receptors that have not yet formed, it also disrupts the dimers of those that have already formed, thus enhancing the anticancer activity (Oliveira et al., 2020). The reversible TKI lapatinib is sensitive and resistant to the proliferation of some cells that lack HER2 gene amplification but carry HER2 somatic mutations. Neratinib can inhibit the growth of these cells (Xu et al., 2017). In addition, neratinib has the ability to reverse multidrug resistance. Furthermore, neratinib is considered to be a sensitive substrate of CYP3A and is eliminated mainly by hepatic metabolism (Yu et al., 2019).

### Afatinib

Afatinib (BIBW2992) is the first oral irreversible EGFR family blocker that can potently inhibit HER2, HER1(EGFR) and HER4. It blocks signal initiated by ErbB family members from all homo- and heterodimers (Harbeck et al., 2016; Hickish et al., 2022). Afatinib monotherapy was approved by the US FDA for the first-line treatment of patients with EGFR-mutant, metastatic non-small-cell lung cancer (NSCLC) in July 2013 (Dungo and Keating, 2013). Afatinib is a transported substrate and inhibitor of the ABC efflux transporters ABCB1 and ABCG2 (van Hoppe et al., 2017). Although afatinib has been approved for NSCLC treatment, it might be also beneficial for other tumors containing the EGFR mutations as well. Preclinical data suggest that afatinib shows activity in HER2+ MBC breast cancer models (Hurvitz et al., 2014; Collins et al., 2021). The IC50 of afatinib required to inhibit EGFR was found to be 1.6 nM (Tu et al., 2016).

The mechanisms of action of several TKIs are shown schematically in Figure 1, and the pharmacological characteristics of three HER2-targeted irreversible TKIs are shown in Table 1.

**TABLE 1:** Pharmacologic characteristics of three irreversible TKIs.

**Drug**	**Pyrotinib**	**Neratinib**	**Afatinib**
Brand name	Irene	Nerlynx	Gilotrif
Targets	HER1; HER2; HER4	HER1; HER2; HER4	HER1, HER2, HER4
IC50(nM)	5.6; 8.1	92; 59; 19	1.6
Route of administration	Oral	oral	oral
Recommended dose	400 mg once daily	240 mg once daily	40 mg once daily
Metabolism	CYP3A4	CYP3A4	ABCB1, ABCG2
Molecular formula	C_32_H_31_CIN_6_O_3_	C_30_H_29_CIN_6_O_3_	C_24_H_25_ClFN_5_O_3_
PubChem CID	51039030	9915743	10184653
3D-chemical structure	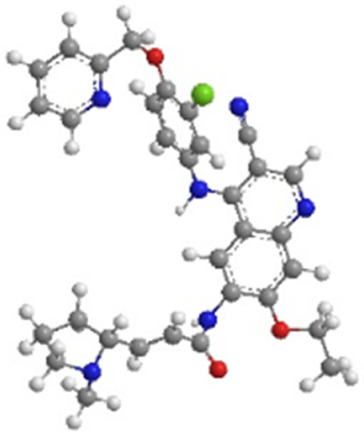	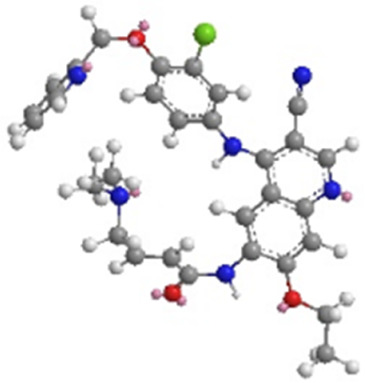	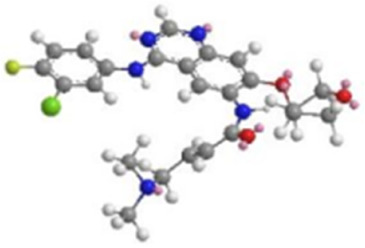

Note: HER, Human epidermal growth factor receptor; TKIs, tyrosine kinase inhibitors.

## Key clinical trials

The mechanisms of action of the three irreversible TKIs were previously briefly described, the paper next reviews a selection of published clinical trials that used irreversible TKIs in combination with chemotherapy for HER2-positive MBC to drive changes in treatment patterns. The basic details of the key clinical trials and common treatment-related adverse events (AEs) (Grade ≥3) are shown in Table 2 and Table3, respectively.

**TABLE 2:** Key clinical trials of irreversible TKIs in combination with chemotherapy.

References	ClinicalTrials.gov	Study type	Sample size	Treatment	mPFS (months)	mOS (months)	ORR (%)	Location
**irreversible TKIs + chemotherapy vs. reversible TKIs + chemotherapy**								
Xu et al. (2021)	NCT03080805	III	267	Pyrotinib + capecitabine vs Lapatinib + capecitabine	12.5 vs 5.6	not reached vs 26.9	67.2 vs 51.5	China
Jacobson (2022)	(PHOEBE)		(134/133)		HR: 0.48 (0.37, 0.63)	HR: 0.69 (0.48, 0.98)		
Ma et al. (2019)	NCT02422199	II	128 (65/63)	Pyrotinib + capecitabine vs Lapatinib + capecitabine	18.1 vs 7.0	not reached vs 29.9	78.5 vs 57.1	China
					HR: 0.36 (0.23, 0.58)			
Xie et al. (2021)	NCT04850625	RCT	224 (92/132)	Pyrotinib + vinorelbine vs Lapatinib + capecitabine	8.3 vs 5.0	not reached	NR	China
					HR: 0.47 (0.34,0.65)			
Yang and Wang (2021)	NR	RCT	164 (68/96)	Pyrotinib + chemotherapy vs Lapatinib + chemotherapy	9.0 vs 6.2	NR	60.3 vs 34.4	China
					HR: 0.58 (0.41, 0.83)			
Saura et al. (2020)	NCT01808573 (NALA)	III	621 (307/314)	Neratinib + capecitabine vs Lapatinib + capecitabine	8.8 vs 6.6	24.0 vs 22.2	32.8 vs 26.7	28 countries
					HR: 0.76 (0.63, 0.93)	HR: 0.88 (0.72, 1.07)		
Martin et al. (2013)	NCT00777101	II	233 (117/116)	Neratinib vs Lapatinib + capecitabine	4.5 vs 6.8	19.7 vs 23.6	29.0 vs 40.5	multinational
					HR: 1.19 (0.89, 1.60)	HR: 1.25 (0.83, 1.86)		
**irreversible TKIs + chemotherapy vs other regimens**								
Yan et al. (2020)	NCT02973737 (PHENIX)	III	279 (185/94)	Pyrotinib + capecitabine vs placebo + capecitabine	11.1 vs 4.1	not reached	68.6 vs 16.0	China
					HR: 0.18 (0.13, 0.26)			
Tian et al. (2022)	NR	RCT	20 (10/10)	Pyrotinib + capecitabine vs capecitabine	18.5 vs 6.5	NR	80.0 vs 40.0	China
Pellerino et al. (2022)	NR	observational study	20 (10/10)	Neratinib + capecitabine vs intrathecal ARA-C/+ WBRT	4.0 vs 1.0	10.0 vs 2.0	30.0 vs NR	Italy
						HR: 0.21		
Cunningham et al. (2022)	Ref. SE768	RCT	72 (45/27)	Neratinib + capecitabine vs Neratinib	7.2 vs 2.9	18.9 vs 7.7	46.6 vs 25.9	London
					HR: 0.38 (0.23, 0.65)	HR: 0.42 (0.24, 0.72)		
Awada et al. (2016)	NCT00915018 (NEfERT-T)	III	479 (242/237)	Neratinib + paclitaxel vs trastuzumab + paclitaxel	12.9 vs 12.9	HR: 1.05 (0.76, 1.45)	74.8 vs 77.6	multinational
					HR: 1.02 (0.81, 1.27)			
Harbeck et al. (2016)	NCT01125566 (LUX-Breast 1)	III	508 (339/169)	Afatinib + vinorelbine vs trastuzumab + vinorelbine	5.5 vs 5.6	20.5 vs 28.6	46.1 vs 47.0	multinational
					HR: 1.10 (0.86, 1.41)	HR: 1.48 (1.12, 1.95)		
Cortés et al. (2015)	NCT01441596 (LUX-Breast 3)	II	81 (38/43)	Afatinib + vinorelbine vs treatment of physician’s choice	12.3 vs 18.4	37·3 vs 52.1	NR	multinational
					HR: 0.94 (0.57, 1.54)	HR: 1.60 (0.93, 2.76)		
**irreversible TKIs + chemotherapy(single-arm)**								
Li et al. (2019)	NCT02361112	I	28	Pyrotinib + capecitabine	22.1 (9.0, 26.2)	NR	78.6	China
Yin et al. (2022)	NR	RWS	172	Pyrotinib + capecitabine	8.8 (6.47, 11.19)	NR	61.0	China
Yan et al. (2022)	NCT03691051 (PERMEATE)	II	78	Pyrotinib + capecitabine	11.3 (7.7, 14.6)	not reached	66.7	China
Hua et al. (2022b)	NCT04899128	RWS	50	Pyrotinib + capecitabine/vinorelbine	8.0 (5.1, 10.9)	not reached	17.1	China
Chen et al. (2020)	NR	RWS	133	Pyrotinib + capecitabine/abraxane	8.7 (7.04, 9.10)	not reached	40.5	China
Zhang et al. (2021)	ChiCTR1900021819	RWS	69	Pyrotinib + capecitabine	15.1 (10.0, 18.8)	not reached	38.6	China
Wang and Huang (2022)	NCT03876587 (PANDORA)	II	79	Pyrotinib + docetaxel	15.0 (10.54, 19.53)	NR	79.2	China
Li et al. (2021)	NCT04517305	retrospective study	97	Pyrotinib + vinorelbine	7.8 (4.7, 10.8)	not reached	34.3	China
Freedman et al. (2019)	NCT01494662 (TBCRC022)	II	49	Neratinib + capecitabine	5.5 (0.8, 18.8)	13.3 (2.2, 27.6)	44.9	multinational
Wang et al. (2021b)	NCT03377387	Ib/II	34	Neratinib + capecitabine	NR	NR	27.3	United States
Saura et al. (2014)	NCT00741260	I/II	105	Neratinib + capecitabine	9.8 (8.2, 16.8)	NR	63.2	multinational
Chow et al. (2013)	NCT00445458	I/II	110	Neratinib + paclitaxel	13.3 (11.1, 19.0)	NR	72.7	multinational
Awada et al. (2013)	NCT00706030	I/II	91	Neratinib + vinorelbine	11.2 (7.2, 15.2)	NR	35.3	multinational
Hickish et al. (2022)	NCT01271725 (LUX-Breast 2)	II	87	Afatinib + vinorelbine/paclitaxel	8.9 (6.9, 12.0)	NR	30.8	Asia & Europe

Note: TKIs, tyrosine kinase inhibitors; mPFS, median progression-free survival; mOS, median overall survival; ORR, objective response rate; RCT, randomized controlled trial; RWS, real-world study; NR, No report; HR, hazard ratio.

**TABLE 3:** Common treatment-related AEs (Grade ≥3) (%).

References	Diarrhea	Hand–foot syndrome	Vomiting	Neutropenia	Leukopenia
**irreversible TKIs + chemotherapy vs. reversible TKIs + chemotherapy**					
Xu et al. (2021)	31.0 vs 8.0	16.0 vs 15.0	6.0 vs 2.0	7.0 vs 5.0	8.0 vs 2.0
Ma et al. (2019)	15.4 vs 4.8	24.6 vs 20.6	4.6 vs 1.6	9.2 vs 3.2	7.7 vs 1.6
Xie et al. (2021)	23.9 vs 8.3	1.1 vs 0	1.1 vs 0.8	7.6 vs 5.3	4.3 vs 7.6
Yang and Wang (2021)	13.8 vs 12.2	3.1 vs 15.6	0 vs 4.4	1.5 vs 3.3	NR
Saura et al. (2020)	24.4 vs 12.5	9.6 vs 11.3	4.0 vs 1.9	NR	NR
Martin et al. (2013)	28.0 vs 10.0	0 vs14.0	4.0 vs 2.0	2.0 vs 4.0	NR
**irreversible TKIs + chemotherapy vs other regimens**					
Yan et al. (2020)	30.8 vs 12.8	15.7 vs 5.3	2.2 vs 1.1	3.8 vs 2.1	3.8 vs 2.1
Tian et al. (2022)	20.0 vs 20.0	10.0 vs 10.0	10.0 vs 10.0	NR	NR
Pellerino et al. (2022)	20.0 vs NR	0 vs NR	10.0 vs.NR	NR	NR
Cunningham et al. (2022)	7.0 vs 11.0	4.4 vs 3.7	8.8 vs2.2	4.0 vs 0	4.0 vs 0
Awada et al. (2016)	30.4 vs 3.8	1.7 vs 3.8	2.5 vs 0.9	13 vs 14.5	8 vs 10.7
Harbeck et al. (2016)	19.0 vs 0	2.0 vs 0	3.0 vs 0	56.0 vs 60.0	19.0 vs 21.0
Cortés et al. (2015)	38.0 vs 10.0	0 vs 0	3.0 vs 0	81.0 vs 21.0	6.0 vs 5.0
**irreversible TKIs + chemotherapy(single-arm)**					
Li et al. (2019)	10.7	3.6	3.6	3.6	3.6
Yin et al. (2022)	16.0	1.3	0.6	9.0	7.1
Yan et al. (2022)	24.0	8.0	2.0	14.0	14.0
Hua et al. (2022b)	14.5	0	1.3	0	NR
Chen et al. (2020)	19.6	6.5	7.7	NR	7.7
Zhang et al. (2021)	4.7	0	0.8	2.4	0
Wang and Huang (2022)	21.5	NR	NR	27.8	29.1
Li et al. (2021)	22.7	NR	1.0	7.2	4.1
Freedman et al. (2019)	29.0	0	4.0	0	0
Wang et al. (2021b)	4.5	0	NR	NR	NR
Saura et al. (2014)	23.0	12.0	0	NR	NR
Chow et al. (2013)	29.0	3.0	2.0	20.0	18.0
Awada et al. (2013)	28.0	NR	39.0	46.0	17.0
Hickish et al. (2022)	8.0	4.0	4.0	38.0	0

Note: TKIs, tyrosine kinase inhibitors; NR, no report.

### Irreversible TKIs plus chemotherapy vs reversible TKIs plus chemotherapy

The comparative efficacy of irreversible TKIs and reversible TKIs is a point of interest for researchers. Five studies comparing the efficacy and safety of irreversible TKIs plus chemotherapy with reversible TKIs plus chemotherapy in patients with HER2-positive MBC have been conducted.

The PHOEBE trial (Xu et al., 2021) (NCT03080805) is a well-known phase-III trial assessing the efficacy of pyrotinib or lapatinib plus capecitabine in patients with HER2-positive MBC who have been previously treated with taxanes and trastuzumab. A total of 267 patients are enrolled. At the time of interim analysis, the results showed that the median PFS (mPFS) was significantly improved in the pyrotinib group compared with the lapatinib group (12.5 vs 6.8 months). The hazard ratio (HR) was 0.39 (95% CI [0.27, 0.56]), which means that the risk of disease progression was reduced by approximately 61% in the pyrotinib group. The most common-treatment-related AEs classified as grade 3 or worse were diarrhea (31% vs 8%) and hand–foot syndrome (16% vs 15%). In the pyrotinib and lapatinib groups, one and two patients had died from AEs, respectively, irrespective of their relations to treatment. In the updated analysis (Jacobson, 2022), the 1-year OS rates for the two groups were 66.6% and 58.8%, respectively. Ma et al. also assessed the efficacy and tolerability of pyrotinib or lapatinib plus capecitabine in HER2-positive MBC patients: the objective response rates (ORRs) for the two arms were 78.5% and 57.1%, respectively, and the mPFS values were 18.1 and 7.0 months (HR, 0.36; *p* < 0.001). T); the most frequent treatment-related AEs were hand–foot syndrome (24.6% vs 20.6%), diarrhea (15.4% vs 4.8%), and a decreased neutrophil count (9.2% vs 3.2%) (Ma et al., 2019). Based on the initial findings from these trials, pyrotinib plus capecitabine significantly improves the PFS and OS in patients with HER2-positive MBC compared with lapatinib plus capecitabine, and both have a manageable level of toxicity. This regimen was approved in China as a second-line treatment for patients with HER2-positive MBC in 2020.

In addition to capecitabine, vinorelbine, taxanes, and gemcitabine are also commonly used chemotherapeutic agents in this combination regimen. A multicenter, retrospective study (Xie et al., 2021) screened 224 patients treated with pyrotinib plus vinorelbine or lapatinib plus capecitabine. The pyrotinib group exhibited a significant improvement in mPFS compared with the lapatinib group (8.3 vs 5.0 months; HR, 0.47; *p* < 0.001), and the mOS was not reached at the time of analysis. The most frequent treatment-related AEs were diarrhea (23.9% vs 8.3%), hand–foot syndrome (0% vs 4.5%), and increased alanine aminotransferase (0% vs 2.3%). Yang and Wang (Yang and Wang, 2021) retrospectively analyzed 164 patients with HER2-positive metastatic or recurrent breast cancer. After unsuccessful first-line trastuzumab treatment, these patients were re-treated with either pyrotinib or lapatinib in combination with chemotherapy. At the time of interim analysis, the pyrotinib group showed a significant improvement in ORR compared with the lapatinib group (60.3% vs 34.4%), and the mPFS was also prolonged (9.0 months vs 6.2 months). The incidence of diarrhea was high in both groups (81.5% vs 72.2%) but was mainly classified as grade 1 to 2, and the incidence of hand–foot syndrome was higher in the lapatinib group (20% vs 46.7%).

The NALA trial (Saura et al., 2020) (NCT01808573) is a multinational phase-III trial comparing the efficacy of neratinib or lapatinib plus capecitabine in 621 patients with HER2-positive MBC previously treated with at least two HER2-targeted regimens. At the cutoff date, the mPFS was significantly improved in the neratinib group, the mean difference being 2.2 months (HR, 0.76; *p* = 0.0059). Although a longer OS was observed in the neratinib arm (24.0 vs 22.2 months), the difference was not statistically significant (HR, 0.88; *p* = 0.2086). The ORRs for the two groups were 32.8% and 26.7%, respectively (*p* = 0.1201). Diarrhea (24.4% vs 12.5%), hand–foot syndrome (9.6% vs 11.3%), and vomiting were the most common AEs. The results of the pan-Asian subgroup analysis in the NALA trial were consistent with the efficacy observed in the overall study population (Dai et al., 2021). Based on the NALA trial, Saura et al. (Saura et al., 2021) explored the correlation between biomarkers and PFS. The results confirmed that both PIK3CA mutations and HER2 expression were associated with PFS, with the former being negatively correlated and the latter positively correlated. Notably, back in 2013, Martin et al. (Martin et al., 2013) compared the efficacy of treatment with neratinib alone compared with the use of lapatinib plus capecitabine in patients with HER2+MBC. The results of this study were considered inconclusive, as the non-inferiority of neratinib was not significant (HR, 1.19). However, it provided preliminary confirmation of the clinical activity and tolerability of neratinib in patients with recurrent HER2-positive MBC.

To further validate the results of the above clinical trials, the combined effect sizes for the primary endpoints mPFS, ORR, and the most frequent grade ≥3 AEs (diarrhea and hand–foot syndrome) were calculated by stata14.0 software for the five RCTs described above. The combined effect size of HR for mPFS was 0.53 (95% CI [0.46, 0.6]; *p* = 0.007) (Supplementary Figure S1), implying a reduction in the risk of disease progression of approximately 47% in the irreversible TKI groups compared with the reversible TKI groups. The combined effect size of HR for mPFS for the four pyrotinib-related studies was 0.47 (95% CI [0.39, 0.55]), which seems to suggest that pyrotinib has better efficacy than neratinib in terms of improving mPFS. The risk ratio (RR) for ORR was 1.31 (95% CI [1.16, 1.49]) (Supplementary Figure S2), meaning that the ORR of the irreversible TKI groups was approximately 31% higher than the ORR of the reversible TKI groups. The RR for diarrhea and hand-foot syndrome (grade ≥3) was 2.29 (95% CI [1.77, 2.95]) and 0.82 (95% CI [0.61, 1.11]), respectively (Supplementary Figures S3, S4). These results suggest that diarrhea is a common adverse event for pyrotinib and neratinib, with an incidence approximately two times higher than that of lapatinib. Numerically, pyrotinib and neratinib are less toxic to the skin of the hands and feet than lapatinib, but there does not appear to be a significant difference.

In summary, the use of irreversible TKIs combined with chemotherapy is more advantageous for the treatment of HER2-positive MBC than reversible TKIs combined with chemotherapy and it has a manageable level of toxicity. The most frequent treatment-related AEs are diarrhea and hand–foot syndrome. Treatment with irreversible TKIs reduces the risk of disease progression by approximately 47% and improves the ORR by approximately 31% compared with reversible TKIs. However, the incidence of diarrhea with these is approximately twice that of reversible TKIs. There is no significant increase in toxicity to the skin of the hands and feet.

## Irreversible TKIs plus chemotherapy vs other regimens

In addition to comparisons with reversible TKIs, some RCTs involving the comparison of irreversible TKIs plus chemotherapy with other regimens were collected. Two trials (Yan et al., 2020; Tian et al., 2022) evaluated the efficacy of treatment with pyrotinib plus capecitabine for HER2-positive MBC, and the data show that patients treated with pyrotinib plus capecitabine had better survival outcomes than those treated with capecitabine alone (mPFS: HR, 0.18; ORR: 68.6% vs 16.0%). In the PHENIX trial (Yan et al., 2020), 71 patients in the placebo group also subsequently received pyrotinib, and the mPFS and ORR were significantly improved. In addition, an *in vitro* test showed that pyrotinib can enhance the radiosensitivity of HER2-positive breast cancer cells (Tian et al., 2022). In line with the previous findings, the most frequent treatment-related AEs for pyrotinib were diarrhea and hand–foot syndrome. Two trials reported that treatment with neratinib plus capecitabine is useful for HER2-positive breast cancer with brain metastases (Cunningham et al., 2022; Pellerino et al., 2022). The results for PFS, OS, and AEs are similar to data from previous trials. Moreover, treatment with neratinib plus capecitabine significantly improves survival outcomes compared with the use of intrathecal Ara-C or whole-brain radiotherapy (WBRT) (Pellerino et al., 2022). Findings from the NEfERT-T (Awada et al., 2016) trial indicate that although the overall efficacy of neratinib-paclitaxel and trastuzumab-paclitaxel is similar, the neratinib-paclitaxel combination may delay central nervous system (CNS) progression.

The LUX-Breast 1 (Harbeck et al., 2016), LUX-Breast 3 (Cortés et al., 2015), and LUX-Breast 2 (Hickish et al., 2022) trials evaluated the efficacy and safety of using afatinib plus vinorelbine for HER2-positive MBC. However, the results show that treatments containing afatinib do not improve patients’ PFS or OS, and the frequency of AEs is high (Table 3). For instance, LUX-Breast 1, an open-label phase-III trial, demonstrated similar mPFS outcomes with afatinib or trastuzumab plus vinorelbine (5.5 vs 5.6 months; HR, 1.10; *p* = 0.43). In other words, afatinib failed to show a significant effect in the treatment of HER2-positive MBC.

Overall, the use of irreversible TKIs, such as pyrotinib or neratinib, in combination with chemotherapy, provides a greater survival benefit than other regimens for patients with HER2-positive MBC, and the data are consistent with the results of previous clinical trials. However, the efficacy of afatinib is yet to be validated. Due to the high heterogeneity of the control group regimens, the combined effect sizes for the primary endpoints were not calculated and compared in this section.

### Single-arm studies of irreversible TKIs plus chemotherapy

Several representative single-arm studies were also summarized here to further demonstrate the efficacy and safety of irreversible TKIs in combination with chemotherapy for HER2-positive MBC, including eight studies on pyrotinib (Li et al., 2019; Chen et al., 2020; Li et al., 2021; Zhang et al., 2021; Hua Y. et al., 2022; Wang and Huang, 2022; Yan et al., 2022; Yin et al., 2022), five studies on neratinib (Awada et al., 2013; Chow et al., 2013; Saura et al., 2014; Freedman et al., 2019; Wang R. et al., 2021), and one study on afatinib (Hickish et al., 2022). Similarly, the pooled effect sizes for mPFS and ORR were statistically analyzed for these single-arm studies. The pooled mPFS values of pyrotinib and neratinib are 10.69 months (95% CI [8.67, 12.71]) and 11.14 months (95% CI [8.84, 13.43]), respectively (Supplementary Figures S5, S6), and the pooled ORRs are 51% (95% CI [37%, 66%]) and 49% (95% CI [32%, 67%]), respectively (Supplementary Figures S7, S8). These results are largely consistent with the previous studies. However, the quality of the evidence is low, as all of these were single-arm studies with small sample sizes.

To summarize, the findings from the abovementioned published clinical trials suggest that irreversible TKIs plus chemotherapy are more beneficial than reversible TKIs plus chemotherapy or other regimens for improving clinical outcomes and survival rates in patients with HER2-positive MBC. The most frequent AEs are diarrhea and hand-foot syndrome, but the incidence of grade ≥3 is low with manageable toxicity levels (Table 3). In addition, the most well-established regimens are pyrotinib or neratinib, in combination with capecitabine or other chemotherapeutic agents such as vincristine and taxanes. In contrast, afatinib failed to show significant activity in the treatment of HER2-positive MBC. In the case of brain metastases, neratinib appears to be superior to pyrotinib. And the activity of irreversible TKIs against brain metastases will be further explored in the next section.

## Activity of irreversible TKIS in brain metastases

**FIGURE 3:**
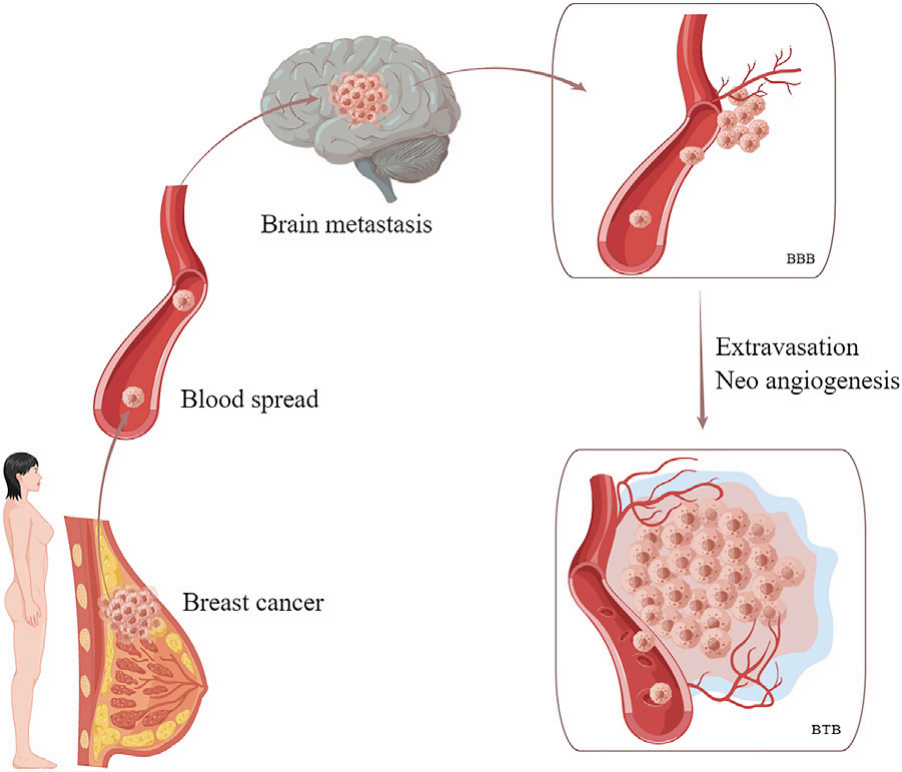
Hematogenous metastasis of breast cancer cells to the brain. Tumor cells cross the BBB and then proliferate around the blood vessels, stimulating neoangiogenesis, disrupting the BBB, and eventually forming the BTB. BBB, blood-brain barrier. BTB, blood tumor barrier.

Brain metastases occur in up to 50% of patients with HER2-positive breast cancers. Due to the low penetration of the CNS, the efficacy of many drugs is limited, resulting in an extremely poor prognosis (Morgan et al., 2021; Schlam and Swain, 2021). Mechanistically, after undergoing epithelial-mesenchymal transition (EMT), breast cancer cells enter the bloodstream where they survive, spread, exude, and implant into the CNS. Tumor cells cross the blood-brain barrier (BBB) and then proliferate around the blood vessels, stimulating neoangiogenesis, disrupting the BBB, and eventually forming the blood tumor barrier (BTB) (Figure 3) (Bailleux et al., 2021; Corti et al., 2022).

Clinical data show that irreversible TKIs can be used against CNS lesions in HER2-positive breast cancers. In a retrospective study, the mPFS of patients with brain metastases in the pyrotinib group was much longer than that in the lapatinib group (6.5 months vs 3.5 months, *p* < 0.05) (Yang and Wang, 2021). Furthermore, the use of pyrotinib together with radiotherapy can significantly improve the PFS, ORR, and duration of response in patients with HER2-positive breast cancer brain metastases without causing serious AEs (Tian et al., 2022). Pellerino et al. (Pellerino et al., 2022) demonstrated that HER2-positive breast cancer patients with leptomeningeal metastases had a 70% probability of receiving a neurological benefit after treatment with neratinib plus capecitabine. Results from the CNS subgroup analysis in NALA (Saura et al., 2020), TBCRC022 (Freedman et al., 2019), and other trials (Hurvitz et al., 2021; Cunningham et al., 2022) also showed that neratinib has an effect on CNS lesions from HER2-positive MBC. The intervention probability for CNS disease was lower in the neratinib group compared to the lapatinib group (cumulative incidence, 22.8% vs 29.2%; *p* = 0.043)11. The mean PFS and OS were significantly longer in the neratinib group than in the lapatinib group (HR: 0.66; 0.90). Among patients with pre-existing CNS lesions at enrolment, the confirmed intracranial ORRs were 26.3% (neratinib plus capecitabine) and 15.4% (lapatinib plus capecitabine) (Hurvitz et al., 2021). The NEfERT-T trial indicated that the neratinib-paclitaxel combination reduced the incidence of CNS recurrences (RR, 0.48; *p* = 0.002) and delayed brain metastasis (HR, 0.45; *p* = 0.004) (Awada et al., 2016).

By contrast, neratinib and pyrotinib appear to be numerically more active than T-DM1 in the brain metastasis population. For instance, the results of the KAMILLA trial, which investigated the efficacy of T-DM1 in treating HER2-positive breast cancer with brain metastases, showed that mPFS, mOS, and ORR were 5.5 (95% CI [5.3, 5.6]) months, 18.9 (95% CI [17.1, 21.3]) months, and 21.4% (95% CI [14.6, 29.6]), respectively (Montemurro et al., 2020). The low response rate to the treatment of brain metastases is due to the presence of BBB and BTB limiting the penetration of anti-tumor drugs into the brain. As previously mentioned, there is a difference in the permeability of the BBB and BTB to small and large molecules, with larger molecule size of anti-HER2 drugs, such as trastuzumab, being more difficult to diffuse into the brain parenchyma than smaller molecule size of TKIs, such as neratinib (Bailleux et al., 2021; Mo et al., 2021). Additionally, neratinib promotes ferroptosis, a non-apoptotic form of cell death, and potently inhibits tumor proliferation and brain metastasis (Nagpal et al., 2019).

In short, irreversible TKIs, particularly neratinib, lead to better survival outcomes for HER-positive breast cancer patients with brain metastases without adding additional AEs. However, there is a lack of basic research on the treatment of brain metastases with irreversible TKIs.

## Discussion

Here, we consider that irreversible pan-HER TKIs may be a promising salvage therapy for patients with HER2-positive MBC after the failure of multiple lines of treatment. According to the results of statistical analysis in our paper, treatment with irreversible TKIs for patients with HER2-positive MBC reduces the risk of disease progression by approximately 47% and improves the ORR by approximately 31% compared with reversible TKIs. Primary or secondary resistance to anti-HER2 therapies is the cause of most treatment failures. Mechanistically, in addition to amplification, HER2 somatic mutation is another mechanism to activate HER2 in breast cancer, with somatic mutations clustered in the extracellular domain of HER2 protein (20%) and the tyrosine kinase domain (68%) (Bose et al., 2013). The mutation sites identified, L755S and del.755-759, are resistant to lapatinib while sensitive to the irreversible inhibitor, neratinib (Bose et al., 2013; Cocco et al., 2018). In addition, irreversible TKIs covalently interact with Cys residues at the ATP binding site and are less likely to develop therapeutic resistance (Chang et al., 2022). Recent studies found that the combination of irreversible pan-HER TKIs with anti-HER2 antibody-drug conjugates (ADCs), such T-DXd, enhanced receptor ubiquitination, which in turn promoted ADCs internalization and efficacy (Li et al., 2020).

With the increasing number of options for beyond-second-line therapies of HER2-positive MBC, several key questions remain. The overriding issues are the efficacy and toxicity of drug combinations. Next is how to select effective TKIs based on the patients’ prior HER2-directed therapies, as there may be cross-resistance. Another issue of note is that brain metastases are a huge challenge for modern oncology, however, there are few reports of CNS-related outcomes in current clinical trials.

Future research should explore new combination therapies, such as the use of HER2-targeted drugs in combination with immune checkpoint inhibitors, PI3K/Akt/mTOR signaling pathway inhibitors, or CDK4/6 inhibitors, which may also lead to more effective and better tolerable treatment options for patients with HER2-positive MBC.

## Conclusion

In summary, irreversible pan-HER TKIs, such as pyrotinib and neratinib, in combination with chemotherapy, represent a beneficial salvage therapy for patients with HER2-positive MBC after the failure of multiple lines of treatment, but their efficacy and safety need to be assessed with more phase-III/IV clinical trials and basic research. In contrast, afatinib failed to show significant activity in the treatment of HER2-positive MBC. Additionally, these irreversible pan-HER TKIs might be promising therapies for patients with brain metastases. Future directions could explore new combination therapies and focus on the safety of cross-dosing.

## Abbreviations

ADCs, antibody-drug conjugates; AEs, adverse events; BC, breast cancer; BBB, blood-brain barrier; BTB, blood tumor barrier; CNS, central nervous system; EGFR, epidermal growth factor receptor; ERK, extracellular signal-regulated kinase; EMT, epithelial-mesenchymal transition; FDA, Food and Drug Administration; HER2, human epidermal growth factor receptor 2; HR, hazard ratio; JAK, Janus kinase; MAPK, mitogen-activated protein kinase; MBC, metastatic breast cancer; mOS, median overall survival; mPFS, median progression-free survival; NSCLC, non-small-cell lung cancerl; NR, no report; ORR, objective response rate; PI3K, phosphoinositide 3-kinase; RWS, real-world study; RCT, randomized controlled trial; SAEs, serious adverse events; STAT, signal transducer and activator of transcription; TKIs, tyrosine kinase inhibitors; T-DM1, trastuzumab emtansine; T-Dxd, trastuzumab deruxtecan; US, United States; WBRT, whole-brain radiotherapy.
