# Supplementary material for: The role of irreversible pan-HER tyrosine kinase inhibitors in the treatment of HER2-Positive metastatic breast cancer
Source: Front Pharmacol. 2023 Mar 2;14:1142087. doi: 10.3389/fphar.2023.1142087 (PMC10018043; doi:10.3389/fphar.2023.1142087)
Supplement: Supplementary file 1 [file DataSheet1.pdf]

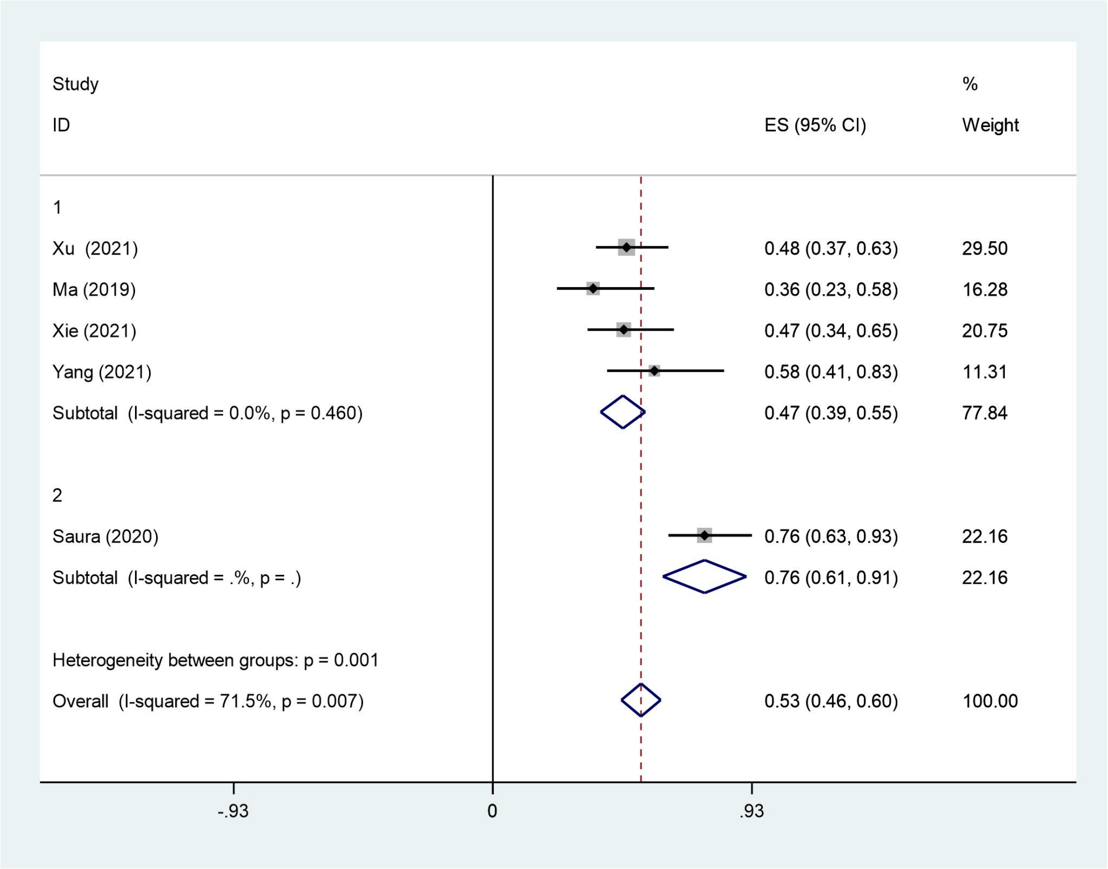

Supplementary Figure S1

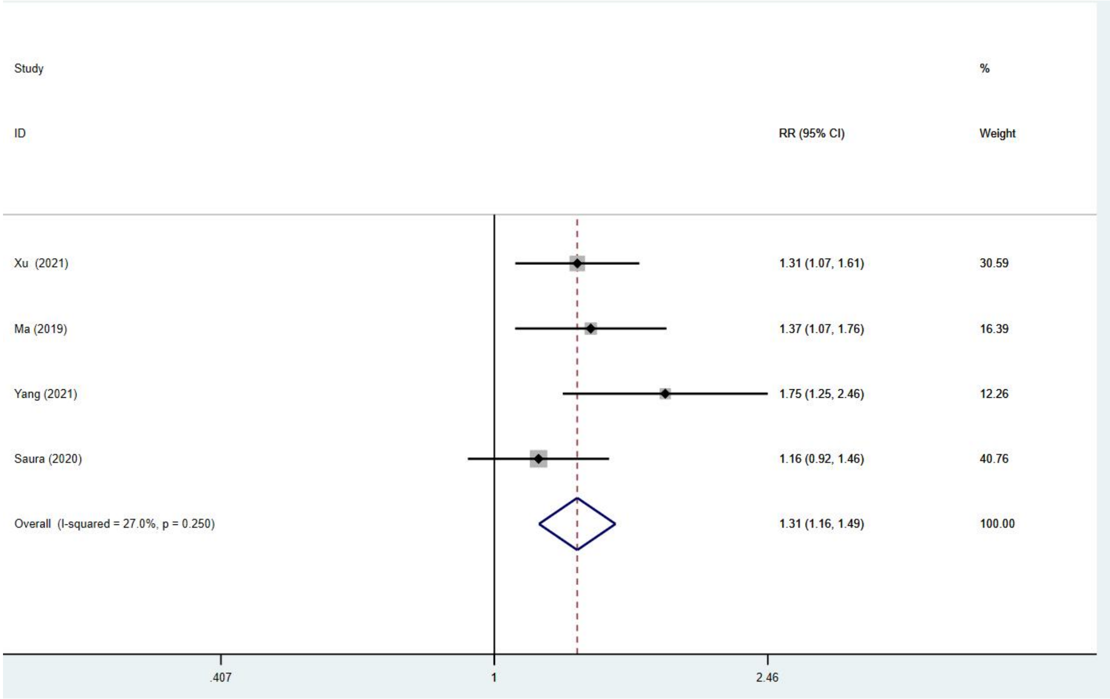

Supplementary Figure S2

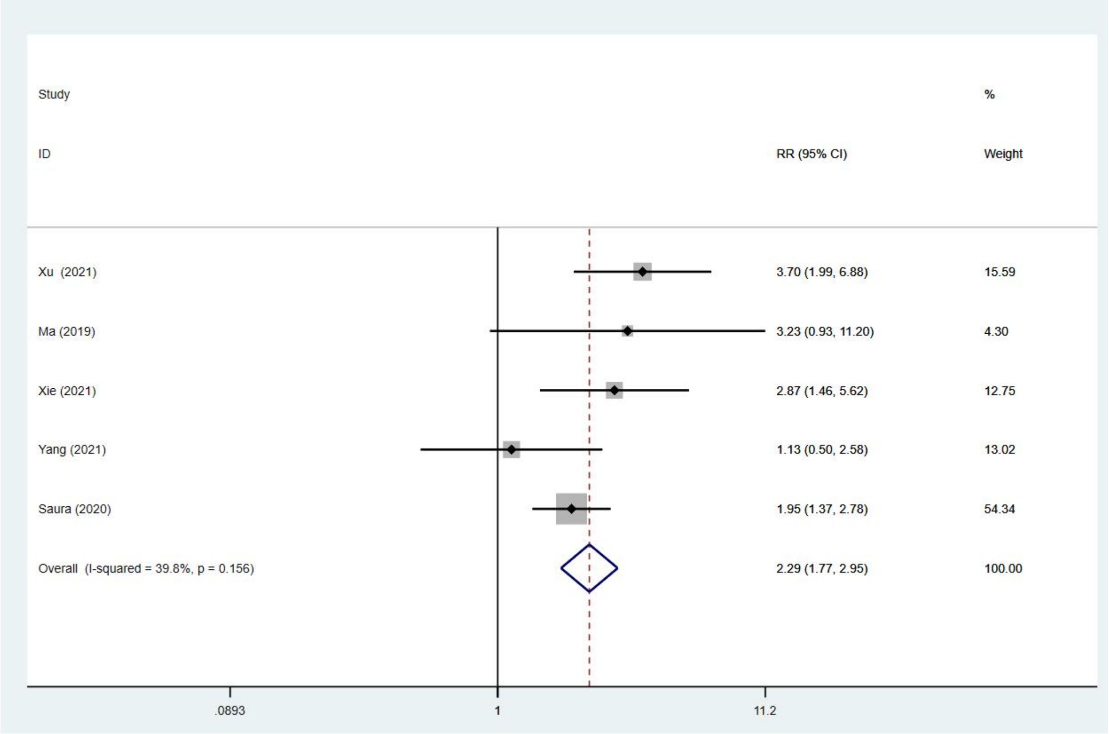

Supplementary Figure S3

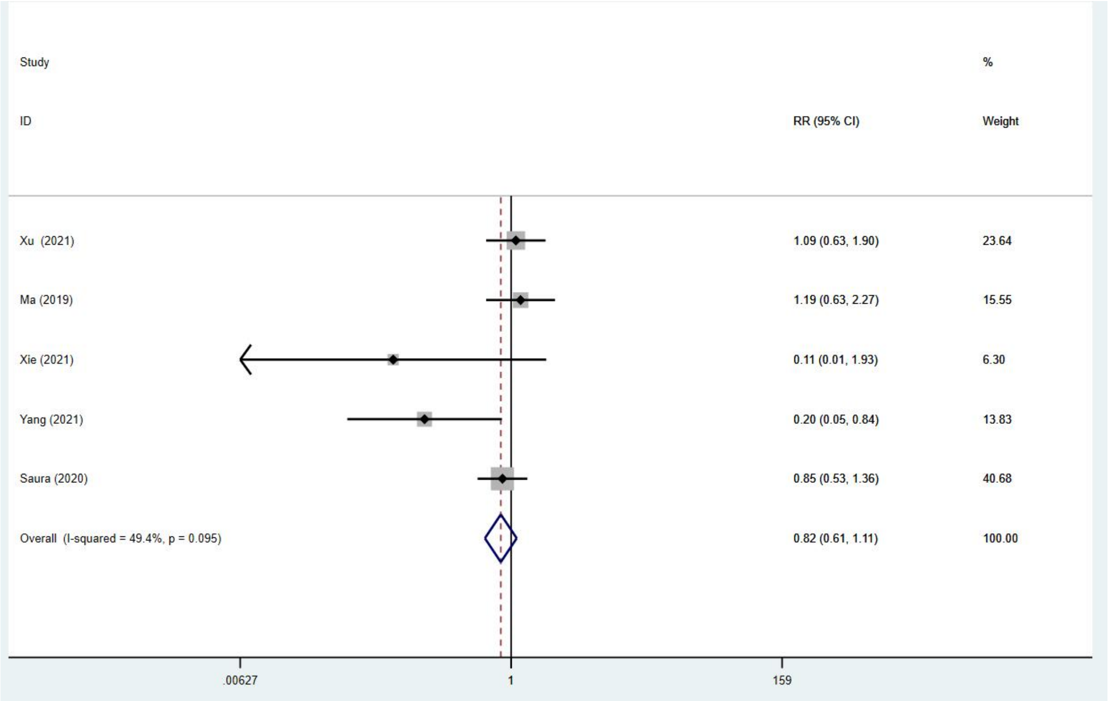

Supplementary Figure S4

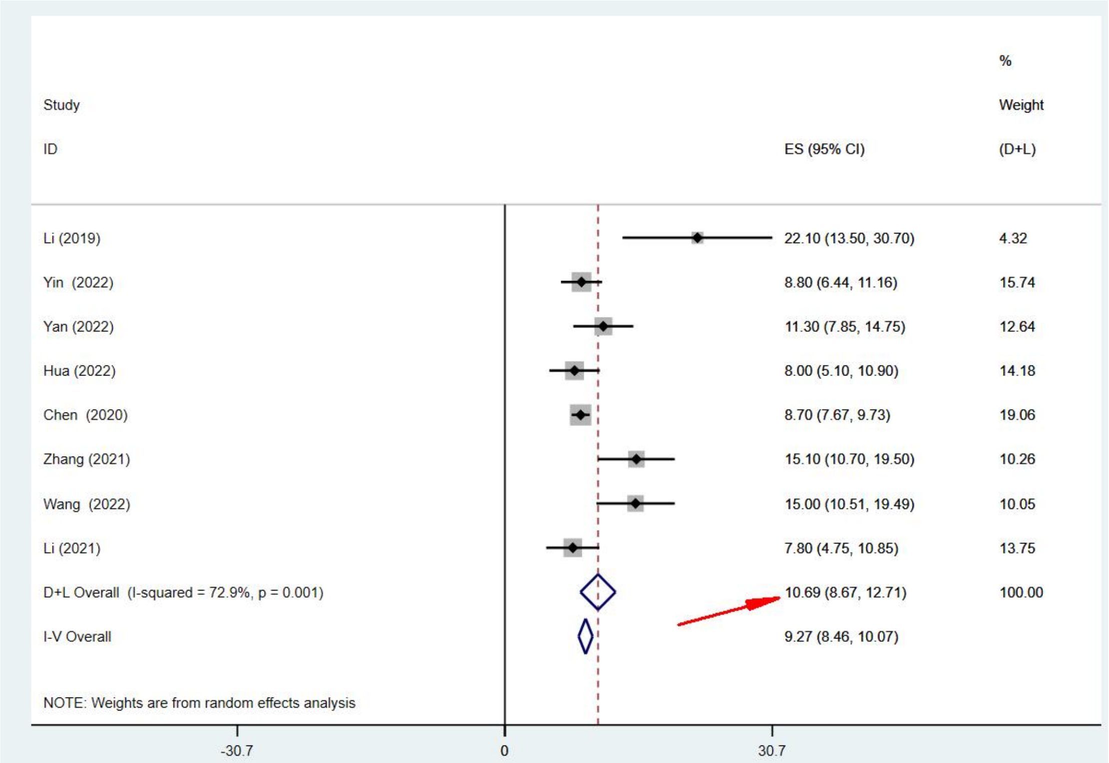

Supplementary Figure S5

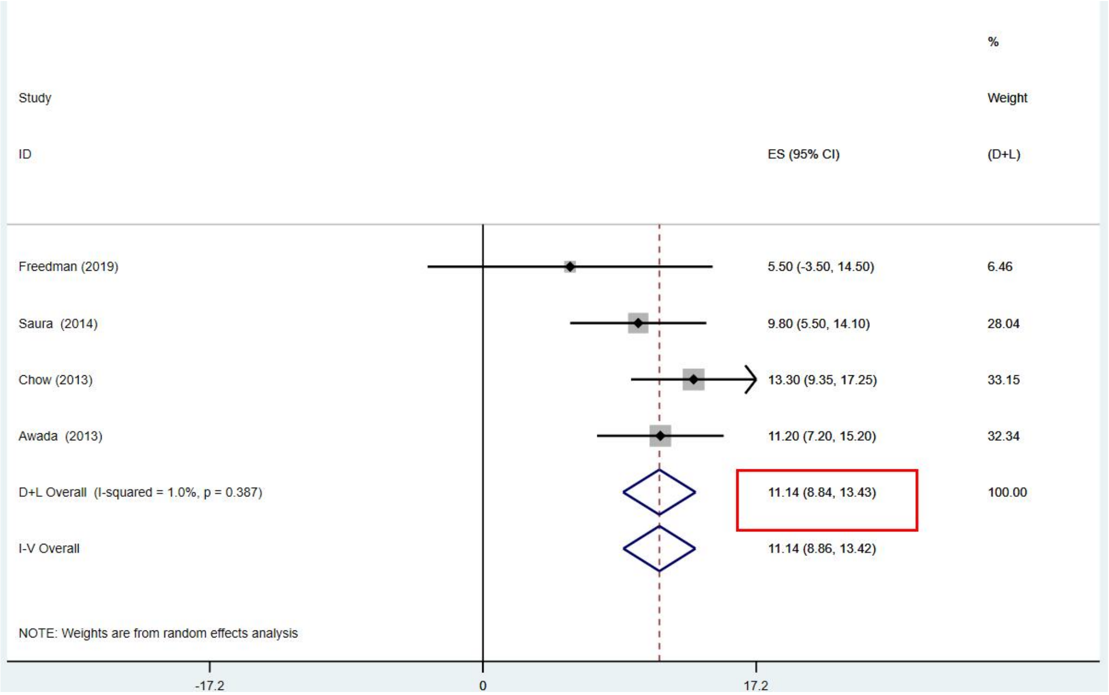

Supplementary Figure S6

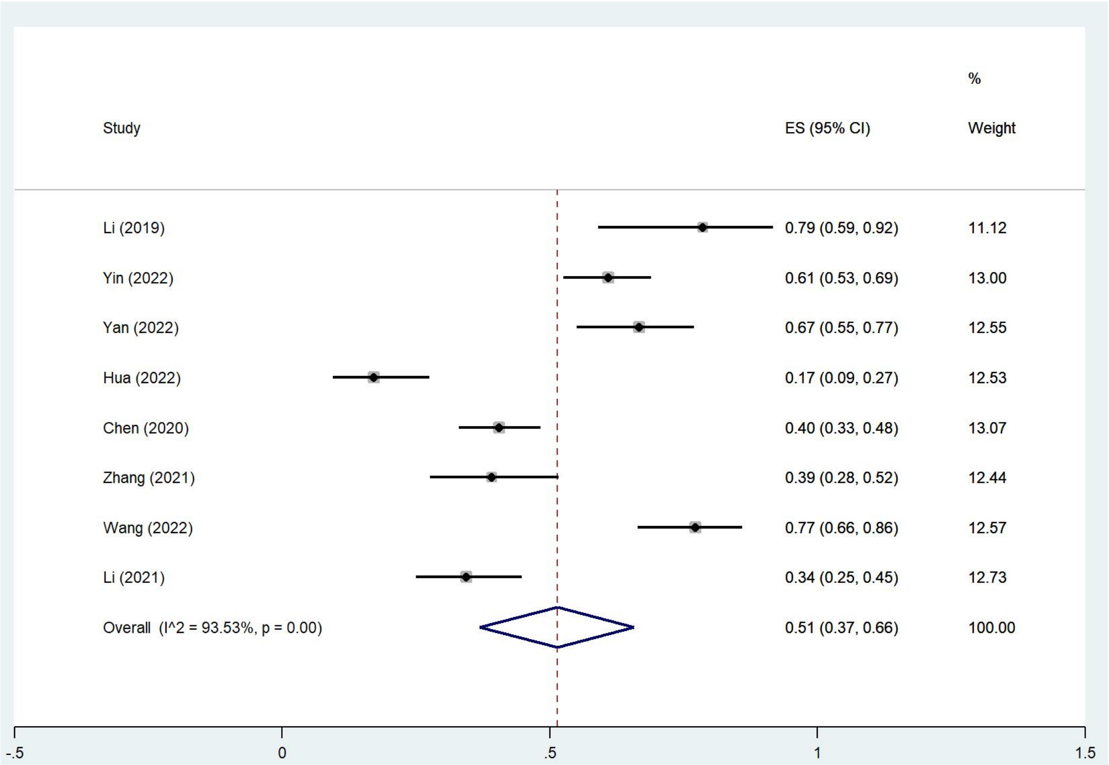

Supplementary Figure S7

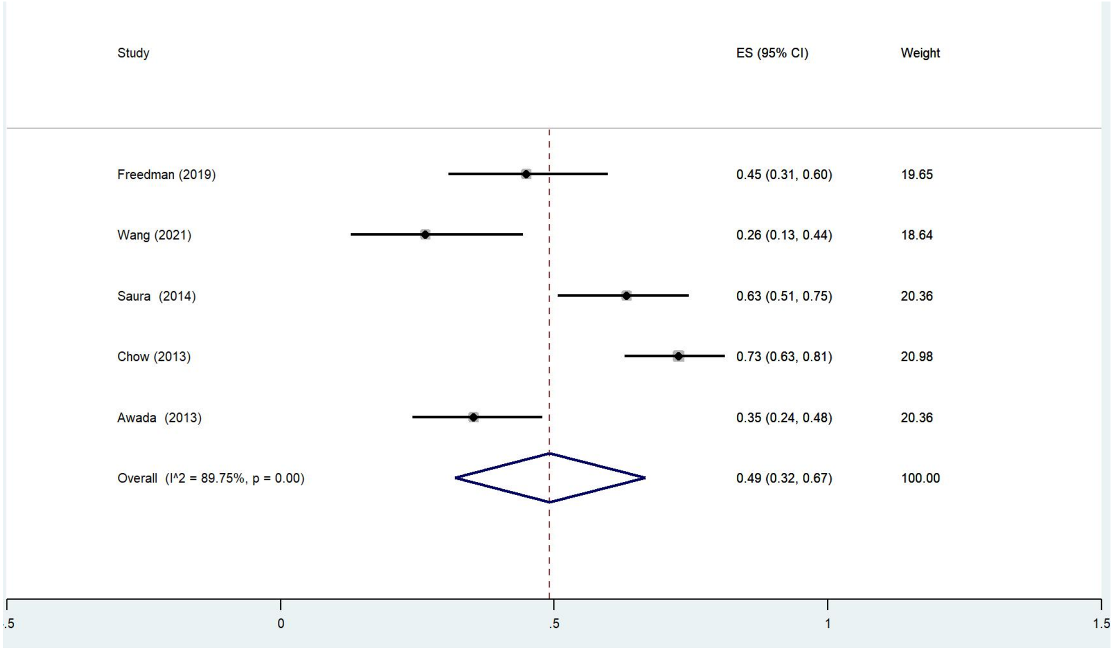

Supplementary Figure S8
